# Erbium-doped (Pb_0.9_-Er_0.01_-Zr_0.09_) and (Pb_0.9_-Er_0.01_-Zr_0.045_-Ti_0.045_) nano-crystalline films and nano-rods ceramic synthesis by sol–gel technique for energy-storage application

**DOI:** 10.1038/s41598-023-31279-3

**Published:** 2023-03-14

**Authors:** F. A. Ibrahim, M. M. El-Desoky

**Affiliations:** 1 Department of Physics, Faculty of Science, Al-Arish University, Al-Arish, Egypt; 2 Academy of Scientific Research and Technology of the Arab Republic of Egypt, Cairo, Egypt; 3 Department of Physics, Faculty of Science, Suez University, Suez, Egypt

**Keywords:** Energy science and technology, Materials science, Nanoscience and technology, Physics

## Abstract

The development of dielectric constant materials for energy storage applications is in high demand. Lead zirconate and lead zirconate titanate doping with erbium thin films and bulk-based devices with variant dielectric constant were created in this work. Pb_(0.9)_-Er_0.01_Zr_(0.09)_ (PEZ) and Pb_0.9_-Er_0.01_-Zr_0.045_-Ti_0.045_ (PEZT) thin films were produced on a glass substrate using a sol–gel doctor blade technique at low temperature. X-ray diffraction (XRD), transmission electron microscopy (TEM), and electron diffraction (ED) were used to examine the structure of the produced nanocrystals. PEZ and PEZT films had nanocrystals that were 9.5 nm and 15 nm in size, respectively, whereas PEZ and PEZT bulk nano-rods had 455 ± 5 nm in length and 45 ± 1 nm in diameter. The TEM and XRD results were found to be completely consistent in terms of particle size. Ferroelectric properties and dielectric characteristics were found to be frequency dependent. Dielectric experiments revealed that the dielectric constant was decreasing for bulk samples compared to film samples. The energy-storage efficiency of PEZ films was roughly 66.01%, and 67.8% for PEZT. The residual polarization of the Er-doped PEZ and PEZT films was the highest, reaching 36.25 μC/cm^2^ and 69.79 μC/cm^2^, respectively, and the coercive fields were 43 kV/cm and 45.43 kV/cm, respectively. On the other hand, PEZ and PEZT bulk samples had residual polarizations of 27.15 μC/cm^2^ and 37.29 μC/cm^2^, respectively, while having coercive fields of 32.3 kV/cm and 39.3 kV/cm, respectively. It was found that (PEZ) and (PEZT) samples may have potential use in energy storage applications.

## Introduction

Local and international researchers have paid close attention to the research and preparation of PZT films over the past years. According to Da Chen et al., ferroelectric PZT thin films have significant applications ranging from transportation systems and electrical power grids to medical devices; such as artificial pacemakers. Because of its outstanding dielectric and ferroelectric characteristics, flexible composition adjustment, and mature preparation technique, lead zirconate titanate (PZT) thin film has been widely utilized in optoelectronics, and other sensors^1,2^. Sputtering, hydrothermal, pulsed laser deposition, chemical vapor deposition, and sol–gel techniques are the most common ways of producing PZT films. Also, the sol–gel technique is now the most popular because of its simple operation, low cost, consistent composition, and ease of element doping application^3–5^. Most researchers have recently concentrated on PZT materials with a Zr/Ti ratio of 52/48. However, changing the Zr/Ti ratio using a single technique cannot fulfill the application requirements of PZT materials^6^. Doping La, Gd, Nd, Nb, and Pr into PZT films can improve their dielectric and ferroelectric characteristics^7–9^. The majority of research has focused on anti-ferroelectric (AFE) bulk ceramics and thin films^10–14^. For example, (PbZrO_3_) (PZ) and (PbZrO_3_TiO_2_) (PZT) films had 35.15 J/cm^3^ and 65.8% for (PZ) and 68.25 J/cm^3^ and 66.6%for (PZT)^1^, while PbZrO_3_ (PZ) films have an energy storage density of 16.8 Jcm^3^ and a 69.2% efficiency^15^. Ceramics are difficult to acquire high energy storage density and are unsuitable for actual energy storage capacitors due to low breakdown strength (BDS), large overall volume, and incompatibility with semiconductor technology^16,17^. It has been observed that element doping is more effective in improving the energy storage properties of AFE materials than undoped films^18,19^. Sr-doped PbZrO_3_ films had an energy-storage density of 14.5 Jcm^3^, which was greater than the 13.3 Jcm^3^ of PbZrO^3^ films^18^. In addition, La^3+^ doped AFE materials have higher stability in the AFE phase^20^, which is beneficial to the energy storage performance.

This research was carried out in order to improve the application of (PEZ) and (PEZT) thin films and bulk in energy storage applications. (PEZ) and (PEZT) thin films and bulk doped with Er concentrations of 0.01% were prepared by the sol–gel technique (doctor blade) to investigate the effect of Er doping on the crystalline structure, dielectric properties, and ferroelectric properties. The concentration of Er doping was adjusted in order to achieve the optimum overall performance from the (PEZ) and (PEZT) films and bulk for dielectric and ferroelectric characteristics. The use of film and bulk improves polarization more than other samples without Er. These results lay the groundwork for the development of high-performance dielectric constants over a wide electronic application range.

## Experimental technique

### Samples preparation and characterization

**Figure 1:**
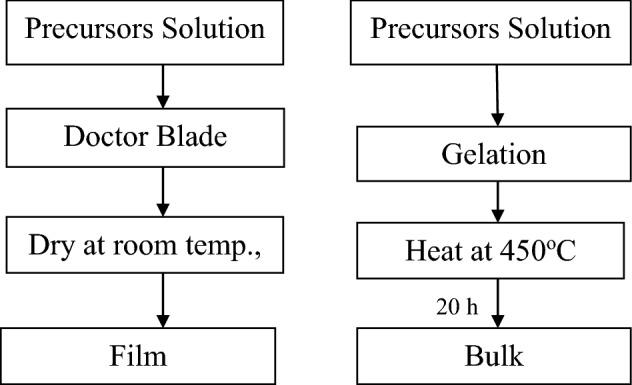
Flow chart for synthesize thin films and bulk samples by sol–gel technique.

Sol–Gel technique was used for preparing the samples (thin films (doctor blade) and bulk) under investigation. Pb_(0.9)_-Zr_(0.09)_ and Er_(0.01)_ (PEZ) and Pb_(0.9)_-Er_(0.01)_-Zr_(0.045)_-Ti_(0.045)_ (PEZT) nanocrystalline thin films and bulk were prepared, using lead oxide (Pb_3_O_4_) with high purity 99% (Sigma Aldrich), zirconium oxide (ZrO_2_), 99% purity (Sigma Aldrich), and titanium dioxide (TiO_2_) 98% purity were selected as raw materials. Erbium nitrate trihydrate (Er(NO_3_)_3_·3H_2_O) 99% purity (Sigma Aldrich) was used as dopant and acetic acid (CH_3_COOH) as solvent for lead oxide. The raw materials of lead oxide (Pb_3_O_4_) were dissolved in acetic acid at 70 °C while stirring. After getting a pure solution, it was cooled to room temperature. Zirconium oxide (ZrO_2_) was dissolved in distilled water at room temperature under stirring, then raising the temperature at 70 °C and erbium nitrate trihydrate (Er(NO_3_)_3_·3H_2_O) was dissolved in distilled water at room temperature without stirring. These three solutions were mixed under stirring at 70 °C for 2 h and aged for 20 h (Fig. 1). Finally, (PEZ) and (PEZT) precursors solution were put directly on substrates by doctor blade technique and then single layer PEZ and PEZT films were prepared by dry process in room temperature. The final thickness of the PEZ and PEZT thin films was about 440 ± 20 nm, measured by the mechanical method (stylus method). The bulk samples were prepared by reaching the solution to the gel stage, then heat treating the gel on a hot plate with a temperature of 100 °C for 1 h for removing moisture from the samples. Finally, the samples were placed in the traditional furnace at a temperature of 450 °C for 20 h, and the PEZ and PEZT bulk were formed by high-temperature sintering and crystallization.

X-ray diffraction (XRD) using a PHILIPS X’Pert diffractometer with a Cu K_α_ target, Ni filter, and 40 kV tube tension in a wide range of Bragg angles.

The morphology of PEZ and PEZT films and bulk were observed by transmission electron microscope (TEM), (Jeol, Jem-1010) in which a TEM observation was performed at an accelerating voltage of 70 kV, while it was just 40 kV in the case of electron diffraction, ED. The dielectric properties of prepared (PEZ) and (PEZT) thin films and bulk were investigated using silver (Ag) paste electrodes. An RLC analyzer (Model 1061, Chen Hwa, LCZ METER, CHINA) was used to assess the frequency dependence of the dielectric properties and dielectric constant/loss versus features of (PEZ) and (PEZT) capacitors (from 40 Hz to 200 kHz at room temperature). A ferroelectric tester (RT1339A, Radiant Technologies) operating at about 20 Hz was used to measure the polarization against electric field (P-E) loops of (PEZ) and (PEZT) capacitors. To rule out any potential hysteretic tendencies for the P-E characteristic, measurements were made as cycle sweeps (zero electric field to positive electric field back to zero electric field, zero electric field to negative electric field back to zero electric field).

## Results and discussion

### PEZ and PEZT thin films and bulk structure properties

**Figure 2:**
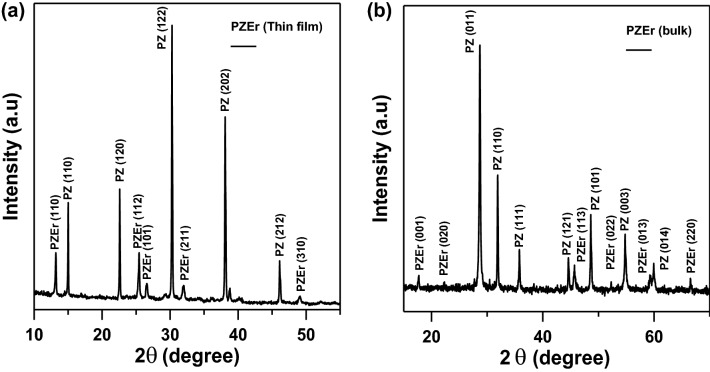
(**a**) XRD pattern of Er-doped PEZ thin film sample. (**b**) XRD pattern of Er-doped PEZ bulk sample.

**Figure 3:**
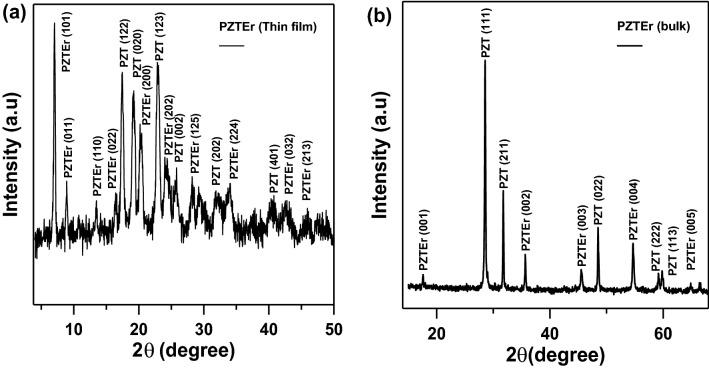
(**a**) XRD pattern of Er-doped PEZT thin film sample. (**b**) XRD pattern of Er-doped PEZT bulk sample.

Figures 2 and 3 show the XRD pattern of PEZ and PEZT thin films and bulk using 40 kV tube tension in the angle range from 5 to 70°. It can be seen from Figs. 2 and 3 that all samples exhibit a nanocrystalline structure with an average grain size of 9.5 nm for PEZ and 15 nm for PEZT by using Sherrer’s equation, which was confirmed by TEM. The Sherrer’s equation that is used to calculate the average particle size, D, is the following^21^:

1 $$D=\frac{k\lambda }{\beta cos\theta }$$

where: θ is the diffraction peak angle of the X-ray, β shows the diffraction peak's corrected (Full-Width at Half Maximum) in radians, k is the shape factor ≅ 1 and λ (nm) is the incident X-ray wavelength (1.5405 Å) of Cu K_α_ radiation. The typical diffraction peaks (110), (120), (122), (202), (212) and (310) correspond to 2θ = 15°, 23°, 30°, 38°, 39°,and 47° for PbZrO_3_ as illustrated in Fig. 2a, in addition, as previously indicated peaks (110), (112), (101), and (211) correspond to 2θ = 25°, 27°, 32°, and 49° for PbZrEr, demonstrate the orthorhombic structure with the JCPDS card No. 01-088-0271 of the thin film. Figure 2b, shows the crystal structure that grows fastest in the high-temperature annealing crystallization process of 450 °C, inhibiting the growth of crystal structures. The diffraction peaks (110), (111), (121), (003), and (014) correspond to 2θ = 33°, 37°, 46°, 55°, and 59° for PbZrO_3_ as shown in Fig. 2b, peaks (001), (020), (113), (022), (013), and (220) correspond to 2θ = 15°, 45°, 53°, 59°, 60°, and 67° for PbZrEr, which illustrate the orthorhombic structure with the JCPDS card No. 98–046-7260 of the bulk sample^22^.

There are two other peaks at 2θ = 29° and 48° corresponding to the diffraction peaks (011) and (112) for PbO in the bulk sample. This implies that the nanocrystalline was effectively created without the presence of any extraneous contaminants. On the other hand, the XRD results for the (Pb_(0.9)_-Er_(0.01)_-Zr_(0.045)_-Ti_(0.045)_) (PEZT) system, Fig. 3, which had a well-developed structure and could be identified by the development of peaks at 7.5°, 9°, 14°, 17.5°, 19°, 21°, 23°, 24°, 26°, 28°, 29°, 32°, 34°, 42°, 44°, and 46° degrees in the (101), (011), (110), (022), (122), (020), (200), (123), (202), (002), (125), (202), (224), (401), (032), and (213) directions for the thin film sample, Fig. 3a. Peak reflections from the film that are in good agreement with the JCPDS (card no. 01-085-0859), which is the same as previously investigated^23–27^, suggest the existence of a tetragonal type structure. The fastest-growing crystal structure in the high-temperature annealing crystallization process, which occurs at 450 °C, is depicted in Fig. 3b, which also shows how this process inhibits the formation of other crystal structures. In addition, peaks (001), (002), (003), (004), and (005) have preferred orientation as they correspond to θ = 17.5°, 36°, 47°, 55°, and 63° for PEZT and illustrate the orthorhombic structure with the JCPDS (card No. 98-006-2849) of the bulk sample^2–6^. (111), (211), (022), (222), and (113) that correspond to θ = 28°, 32°, 49°,59°, and 60° for PZT. The compatibility between the PEZT bulk and the component with each other may promote the growth of PEZT bulk generating this behavior, as demonstrated in Fig. 2 peak density and peak intensity were significantly higher than those of the BEZT film. According to the crystal chemistry principle^24^, Er^3+^ ions enter the Pb and Zr site structures and preferentially replace the Pb^2+^ ions in the Pb sites. Two Er^3+^ ions replace two Pb^2+^ ions or Zr^4+^ ions, creating an O^2−^ vacancy and resulting in a slight contraction of the lattice to preserve electrical neutrality. Furthermore, the ion radius of Er^3+^ (1.01 Å) is smaller than that of Pb^2+^ (1.19 Å)), resulting in a smaller crystal lattice interplaner spacing d^28–30^. The normal growth of grains will be affected because it has been demonstrated that the addition of Er dopant will collect at grain boundaries^2,23^. Some of the Zr^4+^ or Ti^4+^ ions are replaced by Er^3+^ ions when Er doping is added to PEZT. The crystal lattice spacing d increases when the ion radius of Er^3+^ (1.01 Å) increases relative to Zr^4+^ or Ti^4+^ (0.72 Å, 0.61 Å). Additionally, most Er^3+^ ions replace Pb^2+^ ions. However, two Er^3+^ ions must replace two Zr^4+^ or Ti^4+^ ions in order to preserve electrical neutrality, which results in an O_2_ vacancy and a minor contraction of the lattice^29,31^.

**Figure 4:**
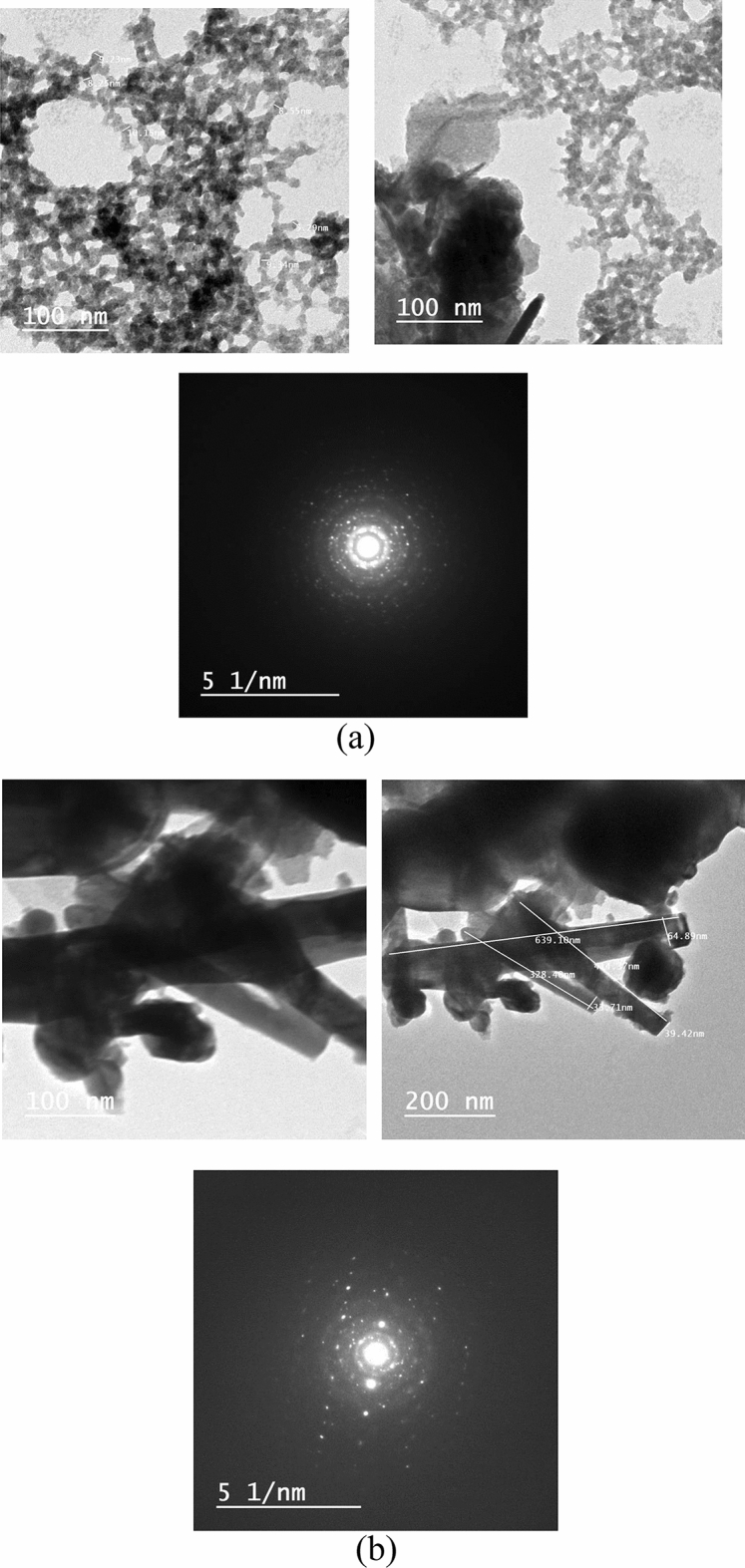
(**a**) TEM of Er-doped PEZ of thin film sample. (**b**) TEM of Er-doped PEZ nano-rods of bulk sample.

**Figure 5:**
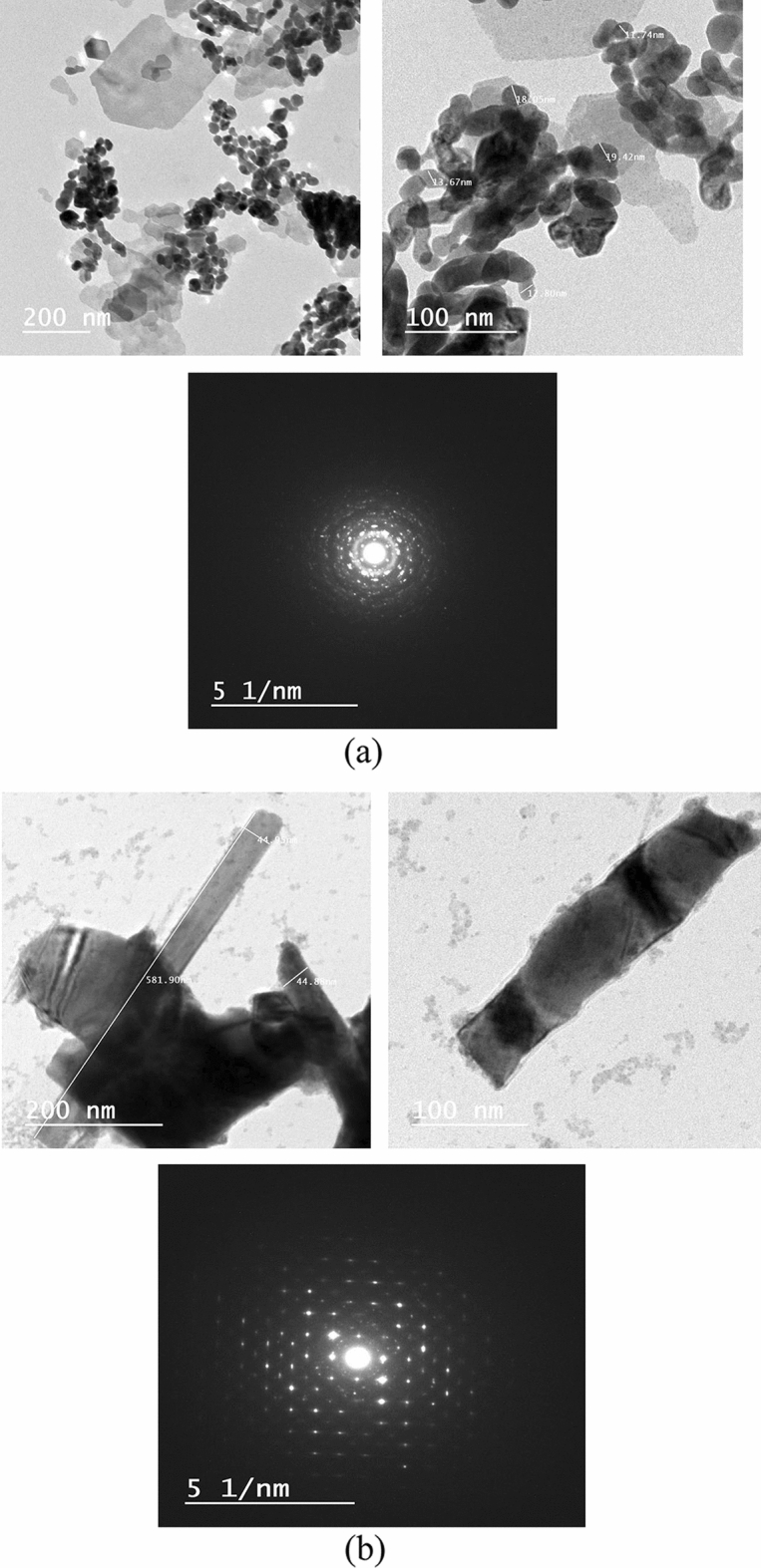
(**a**) TEM of Er-doped PEZT of thin film sample. (**b**) TEM of Er-doped PEZT nano-rods of bulk sample.

The TEM morphology and grain distribution of PEZ samples with thin film and bulk are shown in Fig. 4a,b. Clearly, the nanoparticles in Figs. 4 and 5a are essentially homogeneous in size, and fine grains with an average size of 9.3 nm may be seen in the thin film sample, which is pretty similar to the sizes determined using XRD calculations. Figures 4 and 5b show the nano-rods that have an average diameter of 45 ± 1 nm and 455 ± 5 nm on an average length, hence, are crystalline, according to the TEM results. The crystalline structure was verified by the selected area electron diffraction (SAED) pattern (Fig. 4a,b) for PEZ. The PEZT thin film and bulk sample are photomicrography by TEM and are displayed in Fig. 5a,b. The figure shows that the PEZT film and bulk samples with the doping Er displayed clear nanocrystalline structures. It has been shown that the addition of Er dopant will gather at grain boundaries, which will impair the normal growth of grains, confirmed by XRD^23^. The grains with an average size of 15 nm can be seen in the thin film sample, which is quite similar to the sizes estimated using XRD calculations. According to the TEM measurement, the nano-rods in Fig. 5b are crystalline because they have an average diameter of 45 nm and an average length of 450 nm. The selected area electron diffraction (SAED) pattern proved that the material was crystallized (Fig. 5a,b).

### Dielectric properties

**Figure 6:**
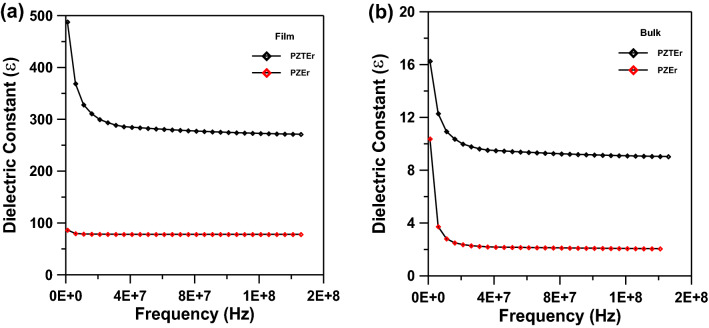
(**a**) Dielectric constant versus frequency for Er-doped PEZ and PEZT thin film samples. (**b**) Dielectric constant versus frequency for Er-doped PEZ and PEZT bulk samples.

Figure 6a,b show the curves of dielectric constant versus frequency of PEZ and PEZT thin films and bulk samples at room temperature. Obviously, the dielectric constant decreases with increasing frequency over the frequency range from 100 Hz to 20 MHz and then begins to remain constant. In general, conductivity effects are related to dielectric constant decreases with frequency. The dielectric constant of PEZ film is about 86.5 and 11 for a bulk sample, but for PEZT film it is about 480 and 17 for a bulk sample. By comparing the dielectric constant of the samples under study (film and bulk), we found that the dielectric constant of the thin film is 8 times larger than the value of the dielectric constant of the bulk sample for PEZ, also for PEZT. This is due to the higher number of dipoles in the thin film than in the bulk form^24,27–30^. The polarization of particles is the greatest contributor to the dielectric constant. The presence of different polarized particles, including space charges, ions, and electrons, is primarily what causes the high dielectric constant at low frequencies. The polarization failure of some particles causes the dielectric constant to decrease, hence, constant as frequency rises^1,2^. Figure 3b shows that the orientation degree increases with the amount of Er-dopant in the PEZT bulk sample. The trends for the dielectric constant and dielectric loss are likewise similar. It's probable that this is the case because that orientation is one that might be used for polarization in the orthorhombic phase, which enhances the dielectric characteristics of PPZT bulk sample as well as the film sample^2^. In general, the dielectric constant and dielectric loss of both film and bulk samples are lower than those of the same composition without erbium (Er) doping and other doped systems for thin film samples^1,2^. A prior study revealed that a dielectric constant is mostly lowered in thin film samples as a result of the polarizability of molecules, which may be varied by modifying the type and amount of polarizable groups^23^. As evidenced by thin film samples, lead vacancy, lattice distortion, local lattice stress reduction, facilitation of domain switching, and an increase in dielectric constant occur when doping Er^3+^ ions replace Pb^2+^ ions as the donors. On the other hand, in bulk samples, Er^3+^ ions take the position of Zr^4+^ or Ti^4+^ ions to play the primary doping role, creating oxygen vacancies. The oxygen vacancy pinning action makes it challenging to flip the domain, and the dielectric constant drops^29,30^. Er dopants assemble on the grain boundary surface at the same time, impeding grain development and lowering the dielectric constant. The switching characteristic of the domain wall, which is comparable to the fluctuation of the dielectric constant, is primarily responsible for the variation of the dielectric loss^31^.

**Figure 7:**
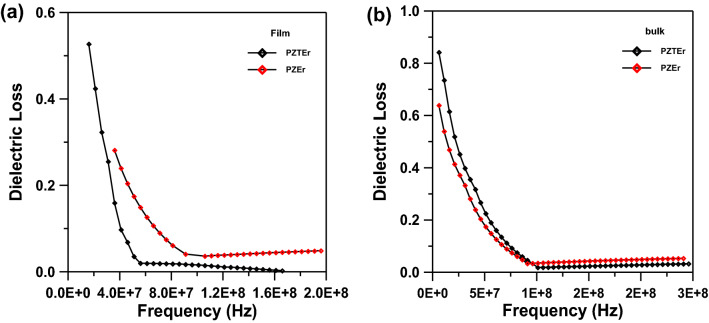
(**a**) Dielectric loss versus frequency for Er-doped PEZ and PEZT thin film samples. (**b**) Dielectric loss versus frequency for Er-doped PEZ and PEZT bulk samples.

The dielectric loss of nanocrystals are shown in Fig. 7a,b, which illustrate the improvement of the dielectric loss by comparing the thin film and bulk samples with other doped and undoped systems^1,2^. The dielectric loss of the thin film sample is smaller than the bulk sample for both PEZ and PEZT. On the other hand, the dielectric loss of the thin film and bulk samples for PEZ is smaller than that of PEZT. This difference is due to the reduced crystal size and crystallinity when the samples are prepared. The dielectric response shows that the produced samples can alter the dielectric constant and dielectric loss. Because the produced film sample lowered crystallinity and made the film more dense by minimizing imperfections, the dielectric loss of the film was lower than that of the bulk sample^29,31–33^.

### Ferroelectric properties

**Figure 8:**
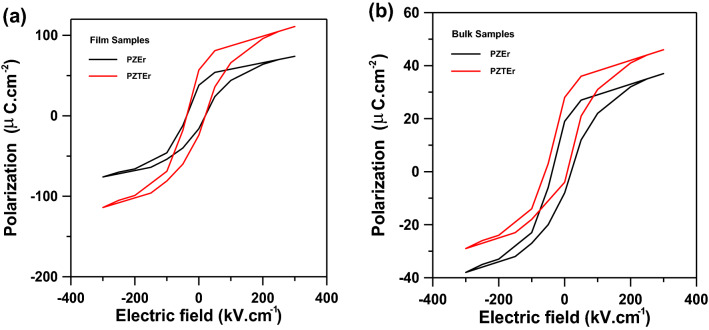
(**a**) P–E hysteresis loops of Er-doped PEZ and PEZT thin films. (**b**) P–E hysteresis loops of Er-doped PEZ and PEZT bulk samples.

Figure 8a,b shows the PEZ and PEZT samples (thin film and bulk) ferroelectric characteristics. When loaded at a frequency of 1000 Hz, Fig. 8a illustrates the polarization hysteresis loops of PEZ and PEZT films with doped Er, respectively. Figure 8a exhibits polarization and electric field direction were asymmetric and saturated polarization hysteresis loops; of the doped PEZ and PEZT films, but Fig. 8b depicts the polarization and electric field direction of the doped PEZ and PEZT bulk samples were both symmetrical, all samples had saturated polarization hysteresis loops. The main cause of the polarization asymmetry, according to a research, was thought to be the buildup of space charges at the electrode-ferroelectric interface. However, the presence of an internal electric field may be the primary cause of the asymmetry in the electric field^4,7^.

It is hypothesized that Er doping modifies the PEZ and PEZT (bulk samples rather than films) internal electric fields, reducing the influence of space charge accumulation at the electrode-PZT interface and enhancing the asymmetry of the polarization hysteresis curve. As seen in Fig. 8a, the remnant polarization and coercive field of PEZ and PEZT thin films first increase and then decrease. The coercive fields of the PEZ and PEZT films were 43 kV/cm and 45.43 kV/cm respectively, while the Er-doped PEZ and PEZT films achieved the largest residual polarization of PEZ and PEZT films, reaching 36.25 μC/cm^2^ and 69.79 μC/cm^2^ respectively. It may be explained that lead vacancies in PEZ and PEZT thin films, when doped with Er, can encourage domain inversion, boost residual polarization, and raise coercive fields. The greatest residual polarization of PEZ and PEZT bulk samples, however, was achieved by the Er-doped PEZ and PEZT bulk samples, which had residual polarizations of 27.15 μC/cm^2^ and 37.29 μC/cm^2^, respectively, while PEZ and PEZT bulk samples have coercive fields of 32.3 kV/cm and 39.3 kV/cm, respectively. However, when doping Er, the oxygen vacancies in the PPZT thin films rise, and the space charges associated to the oxygen vacancy produce a pinning effect to prevent the domain inversion, reducing the remnant polarization and coercive field^7,29,30^.

### Performance of energy storage

During the phase transition of FE materials, the process of polarization switching was always accompanied by a change in the electric field, temperature, or hydrostatic pressure. Therefore, a wide range of applications for FE materials were available, with high energy capacitors ranking as one of the most significant^31,32^. Linear dielectrics and FE materials may be used in electric energy-storage capacitors. Dielectric materials' polarization versus applied electric field (P-E) loops are used to assess their capacity for storing energy. According to the description of energy-storage density via P-E loops, the amount of energy stored in a dielectric per unit volume is determined by the following formula^1,34^:

2 $$ {\text{W = }}\smallint {\text{EdP}} $$

To obtain the value of W based on equation, the area between the polarization axis and the curves of the P-E loops is numerically integrated (2). The investigation showed that the energy-storage density and efficiency for thin films and ceramics were, on average, greater than what was first reported for PZ and PZT thin films^1,3^. The energy-storage density and efficiency for thin films and ceramics were averaging around 36.25 J/cm^3^ for thin films (PEZ) and 69.79 J/cm^3^ for thin films (PEZT) (Fig. 8a). The energy-storage effectiveness (η) and energy-loss density W (loss) were desired for practical applications. P-E loops could also be used to measure the energy-loss density W (loss), and computations revealed that W (loss) for PEZ and PEZT was roughly 18.66 J/cm^3^ and 33.22 J/cm^3^, respectively. With the use of this formula, the energy-storage efficiency (η) might be determined.

3 $$ \left( \upeta \right) \, = {\text{ W}}/\left( {{\text{W }} + {\text{ W}}_{{{\text{loss}}}} } \right) $$

The energy-storage efficiency of PEZ films was roughly 66.01% using the equation above, and 67.8% for PEZT. Due to their high energy density and efficiency, PEZ and PEZT thin films and ceramics were discovered to have a potential utility in the energy storage device. According to the study, the polarization asymmetry was thought to be mostly caused by the buildup of space charges at the electrode-ferroelectric contact. However, the presence of an internal electric field may be the main cause of the asymmetry in the electric field^7,9,31,32^. By altering the internal electric fields of PEZ and PEZT (ceramic samples rather than films), Er doping is anticipated to reduce the effect of space charge accumulation at the electrode-PZT interface and increase the asymmetry of the polarization hysteresis curve. As shown in Fig. 8, the remnant polarization and coercive field of the PEZ and PEZT thin films initially increase before decreasing Fig. 8a. When compared to the PEZ and PEZT thin films, the residual polarization of the Er-doped PEZ and PEZT thin films was found to be 36.25 μC/cm^2^ and 69.79 μC/cm^2^, respectively. The PEZ and PEZT films have coercive fields of 32.3 kV/cm and 39.3 kV/cm, respectively. It might be argued that lead vacancies in Er-doped PEZ and PEZT thin films can enhance coercive fields, promote domain inversion, and increase residual polarization^35–41^.

## Conclusion

The structure and dielectric constant of the thin films and bulk samples are investigated. The findings suggested that the structural, ferroelectric, and dielectric properties of materials affected the erbium content. The structures of the samples are characterized by TEM and XRD. PEZ and PEZT bulk nano-rods had dimensions of 455 nm in length and 45 nm in diameter, whereas PEZ and PEZT films had nanocrystals that were, respectively, 9.5 nm and 15 nm in size, which was confirmed by the results of the TEM. The results of the dielectric properties reveal that samples under study show excellent performances, which could not only obtain a higher dielectric constant but also low dielectric loss for thin films compared to bulk samples. PEZT film has a dielectric constant of roughly 480 and 17 for bulk samples, compared to about 86.5 and 11 for PEZ film. We discovered that, for PEZ and PEZT, the dielectric constant of the thin film is very nearly larger than the value of the dielectric constant of the bulk sample. The Er-doped PEZ and PEZT films had the highest residual polarization, measuring 36.25 μC/cm^2^ and 69.79 μC/cm^2^, respectively, and coercive fields of 43 kV/cm and 45.43 kV/cm, respectively. In contrast, the residual polarizations of the PEZ and PEZT bulk samples were 27.15 μC/cm^2^ and 37.29 μC/cm^2^, respectively, whereas the coercive fields were 32.3 kV/cm and 39.3 kV/cm, respectively. The energy-storage efficiency of PEZ films was roughly 66.01%, and 67.8% for PEZT. As a result, PEZ and PEZT bulk and thin films made using the sol–gel method have good structural qualities, dielectric properties, and ferroelectric properties that can be applied to ferroelectric memory and energy storage applications.

## Data Availability

The datasets used and/or analyzed during the current study available from the corresponding author on reasonable request.
